# The time series seasonal patterns of dengue fever and associated weather variables in Bangkok (2003-2017)

**DOI:** 10.1186/s12879-020-4902-6

**Published:** 2020-03-12

**Authors:** Sittisede Polwiang

**Affiliations:** 1 Department of Mathematics, Faculty of Science, Silpakorn University, Nakhon Pathom, 73000 Thailand; 2 The Center of Excellence in Mathematics, CHE, Bangkok, 10400 Thailand

**Keywords:** Dengue fever, Artificial neuron network, Poisson regression, ARIMA

## Abstract

**Background:**

In Thailand, dengue fever is one of the most well-known public health problems. The objective of this study was to examine the epidemiology of dengue and determine the seasonal pattern of dengue and its associate to climate factors in Bangkok, Thailand, from 2003 to 2017.

**Methods:**

The dengue cases in Bangkok were collected monthly during the study period. The time-series data were extracted into the trend, seasonal, and random components using the seasonal decomposition procedure based on loess. The Spearman correlation analysis and artificial neuron network (ANN) were used to determine the association between climate variables (humidity, temperature, and rainfall) and dengue cases in Bangkok.

**Results:**

The seasonal-decomposition procedure showed that the seasonal component was weaker than the trend component for dengue cases during the study period. The Spearman correlation analysis showed that rainfall and humidity played a role in dengue transmission with correlation efficiency equal to 0.396 and 0.388, respectively. ANN showed that precipitation was the most crucial factor. The time series multivariate Poisson regression model revealed that increasing 1% of rainfall corresponded to an increase of 3.3% in the dengue cases in Bangkok. There were three models employed to forecast the dengue case, multivariate Poisson regression, ANN, and ARIMA. Each model displayed different accuracy, and multivariate Poisson regression was the most accurate approach in this study.

**Conclusion:**

This work demonstrates the significance of weather in dengue transmission in Bangkok and compares the accuracy of the different mathematical approaches to predict the dengue case. A single model may insufficient to forecast precisely a dengue outbreak, and climate factor may not only indicator of dengue transmissibility.

## Background

Dengue fever is one of the most common infectious diseases in Thailand and one of the top threats to global public health. Dengue virus is the cause of dengue fever. The dengue virus is a single positive-stranded RNA virus of the family *Flaviviridae*; genus *Flavivirus*. Approximately a third of the world population are living in dengue-endemic areas, the significant disease burden being in tropical and subtropical regions, which are mostly developing countries [1]. The symptoms of dengue fever individuals range from no signs, mild fever, high fever, pain behind eyes, headache, vomiting, and muscle pains [2]. Severe cases can be massive bleeding, shock, and death. Dengue symptoms can be classified into three categories depending on the clinical syndromes, from mild to severe, dengue fever (DF), dengue hemorrhagic fever (DHF) and dengue shock syndrome (DSS). Dengue virus has four different serotypes (DENV 1-4) that can transmit to humans [3]. Recovery from infection (primary infection) by one serotype provides lifelong immunity against that serotype and temporary for the other. If persons get infected with different serotypes (secondary infection), the risk of developing severe dengue is increasing.

The mosquito, *Ae. aegypti* and *Ae. albopictus*, are the main vector of the dengue virus and mainly feed on human blood [1]. *Ae. aegypti* habit in urban areas while *Ae. albopictus* is in rural areas. There is no specific treatment for dengue fever. The control methods are mainly surveillance and elimination of mosquito. A commercial dengue vaccine, known as CYT-TDV or Dengvaxia, is available in some countries for people ages 9-45 years old. However, the World Health Organization suggests that the vaccine only be provided to persons who have exposures previously to dengue virus [4]. The number of Dengue cases is likely to increase in the future because of several factors such as climate change, globalization, development of the virus, insufficient political and economic supports, and limited resources for effective control measures.

In Thailand, the first report of dengue infection in the country was around 1949, and the first outbreak was 1958 [5]. The recent reports indicated that the significant dengue-endemic occurs typically every 3-5 years [5]. In the last decades, The Bureau of Epidemiology reported that approximately 40,000-150,000 dengue cases per year [5]. Kongsin et al. [6] estimated the total annual economic burden of dengue in Thailand was 125-191 million US dollars, which approximately 72% was the cost of dengue illness and 28% was dengue control programs. In general, the patterns and epidemiological characteristics of dengue mostly depend on climate factors. Humidity, temperature, and rainfall are the key factors [7, 8]. Phanitchat et al. [9] reported that the dengue outbreaks coincide with the rainy season and maximum temperature in Khonkean, Thailand. However, the patterns of dengue incidence also depend on several factors, such as population density, human movement, sanitation, and infrastructure. It is essential to understand the pattern of dengue incidence because it may assist the authorities to prepare and prevent the outbreak.

The objective of this study was to investigate the epidemiological pattern of dengue incidence in Bangkok, Thailand, and also the effects of climate on dengue infection by using the data from 2003-2017 and the mathematical approach. The time-series models can evaluate trends and seasonal patterns of dengue incidence and may apply to predict future endemics. The seasonal-decomposition procedure based on loess (STL) was employed to assess the trend and seasonality of dengue fever. It is essential to use more than one approach to predict dengue cases. In this study, we used three different methods; Multivariate Poisson Regression model (MPR), Artificial Neural Networks (ANN), and Autoregressive Integrated Moving Average (ARIMA). Various studies [10–14] used these methods to predict the dengue-endemics. MPR uses climate factors as a dependent variable and number of dengue cases as an independent variable. ANN uses combinations of independent variables (climate factors) to calculate relationships with dependent variables (dengue cases). ARIMA is a generalization of an autoregressive moving average model and provides another approach to time series forecasting. We used RStudio to stimulated and calculated the results. A high dengue incidence rate typically occurs every 3-5 years [5]. A period of 15 years would be sufficient to obtain the pattern of dengue epidemiology.

## Methods

### Study area

Bangkok is the capital city of Thailand and the most populous in the country. The city is the center for transportation, industry, finance, tourism, education, and trade. The register population was 5.6 million in 2017, and the population density was approximately 3500 per square kilometer. In 2003-2017, the mean temperature was 29.8 ^∘^C, average relative humidity was 72.9%, and the average monthly rainfall was 150 mm.

### Data collection

The Bureau of Epidemiology (BoE), Department of Disease Control, Ministry of Public Health of Thailand, provided Dengue statistical data [5]. The local health services submit the data to the central administration. The BoE published the data on its website and within the Annual Epidemiological Surveillance Reports (AESRs). The dengue data from Thai national surveillance are published monthly. The data consist of the number of dengue cases, fatality, age, and type of dengue. The dengue incidence number data set in this study can be found in the Supplementary file, data set sheet, Table S4.

The Department of Meteorology, Ministry of the Digital Economy and Society, provided the climate data from 2003-2017 [15]. The data set consists of 180 monthly measurements or sets of mean temperature, mean relative humidity, total rainfall, and the number of dengue cases. The climate data set in this study can be found in the Supplementary file, data set sheet, Table S1–S3.

### Mathematical analysis

#### Decomposition

Several types of research include the natural sciences, environmental science, and public health use the seasonal-decomposition procedure based on loess (STL) to analyse the time-series data. SLT filters the trend and seasonal component from the time series data and decomposes into three components: trend (the long term and low-frequency variation in the data), seasonal (variation in the data within the same period), and random or remainder (the remaining variation in the data after extracting trend and seasonal component). The advantages of SLT are its simplicity, robustness of results, and effective data visualization. The time-series data, the trend, seasonal and random component were denoted by *Y*_*t*_, *T*_*t*_, *S*_*t*_ and *R*_*t*_, respectively. The equation can be described as follows.

1 $$ Y_{t}=T_{t}+S_{t}+R_{t}  $$

In this study, *Y*_*t*_ is the number of dengue cases. *t* is time in the unit of month. The numbers of dengue cases are vastly different each year. In the outbreak year, the numbers may triple that of an average number of the whole period. Therefore, it might lead to mistranslate the pattern. It is essential to adjust the numbers of dengue cases each year to the same magnitude. Consequently, we set up the new parameter, adjusted dengue data, $Y^{*}_{t}$, which is defined as follows.

2 $$ Y^{*}_{t}=\frac{Y_{t}}{Y_{max}}  $$

where *Y*_*max*_ is a dengue case of the peak month of the year. We assumed that the period of the dengue-endemic was 12 months; start from January to December. The adjusted value allows us to investigate the pattern of the dengue incidence by reducing the effects of outlier cases.

The variance of *Y*_*t*_ can be described as follows;

3 $$\begin{array}{*{20}l} \text{Var}(Y_{t})=&\text{Var}(T_{t})+\text{Var}(S_{t})+\text{Var}(R_{t})+\text{Cov}(T_{t},S_{t})\\&+\text{Cov}(T_{t},R_{t})+\text{Cov}(S_{t},R_{t}) \end{array} $$

The ratio of the variance of component and the variance of data set was calculated as follows;

4 $$ r=\frac{\text{Var}(C_{t})}{\text{Var}(Y_{t})}  $$

where *r* is the value of the ratio, and *C*_*t*_ is the component of seasonal decomposition. If *r* is close to one, the component is the most important to the data set.

#### Multivariate poisson regression (MPR)

In this study, we applied the Spearman correlation analysis to identify the relationship between the number of dengue cases and mean temperature, rainfall, and humidity with three-month lags in Bangkok. The three-month length is sufficient to cover the life span of the mosquito, incubation, and infectious period of the dengue virus in the human body. We established a time-series Poisson regression model to determine the association between climate factors and dengue cases in Bangkok. Typically, the Multivariate Poisson Regression model expresses the natural logarithm of the outcome as a linear function of a set of predictors can be described as follows;

5 $$ \ln(Y_{t})=\beta_{0}+\sum\limits_{i=1}^{n} \beta_{i}x_{i}+cY_{t-1}  $$

where ln(*Y*_*t*_) is the natural logarithm of predicted dengue cases at time *t*; *β*_0_, *β*_*i*_ and *c* are the constant. *x*_*i*_ represent climate variables.

#### Artificial neural networks (ANN)

The ANN models consisted of three layers; input layer, hidden layer, and output layer. The key advantages of this procedure are ANN can manage a large number of data sets, extract complex nonlinear relationships, and detect interactions between dependent and independent variables. The network model consisted of four parameters in its input layer, namely rainfall, relative humidity, mean temperature, and the number of dengue cases reported last month. The output was the number of dengue cases.

#### The Arima models

An autoregressive integrated moving average (ARIMA) model is a statistical analysis model that uses the time series data to forecasts the possible outcome. A non-seasonal ARIMA model is denoted ARIMA (*p*,*d*,*q*). The non-negative integers, *p* is the number of autoregressive terms. *d* is the number of times that the raw observations are differenced. *q* is the number of lagged forecast errors in the prediction equation. An extension of ARIMA models with the seasonal component is SARIMA (*p*,*d*,*q*)(*P*,*D*,*Q*)^*m*^, where *m* is the number of periods in each season, and *P*,*D*,*Q* are the autoregressive, differencing, and moving average terms for the seasonal part of the ARIMA model respectively.

The data set is divided into two different subsets called train and test set. The main difference between train and test sets is that the train data set is used in training the neural networks, and the test data set is the unseen data that is hidden to the network during training. In this study, total data covered 15 years or 180 months period. The training data set was 168 months period, and the rest 12 months were test set and used to verify the accuracy of the model. The ratio of the selection in the number of data was 0.93 (168/180), which means an excellent validation if trained and successfully tested since the training data set contains less data than the testing set.

## Results

**Fig. 1:**
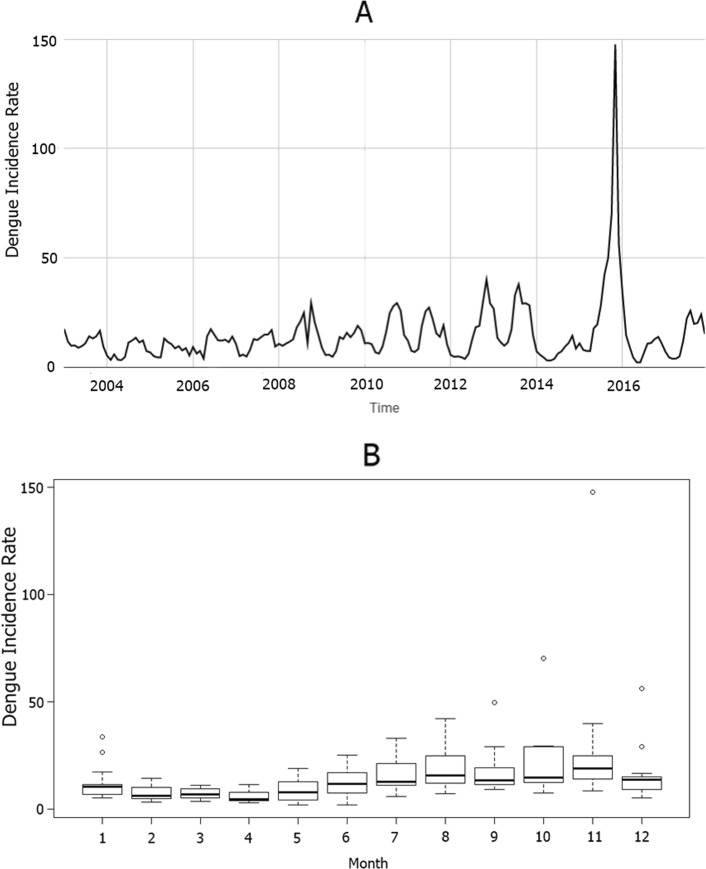
**a**: The number of dengue incidence rate per 100,000 population in Bangkok from 2003 to 2017. **b**: Monthly box plot distribution of dengue incidence rate

Figure 1a shows the reported dengue incidence rate in Bangkok during the study period (2003-2017). The peak of dengue-endemic occurred in November 2015, in which the incidence rate was 147 per 100,000 and also the highest dengue-endemic year, with an incidence rate of 461 per 100,000. The lowest incidence rate was 2014 (83 per 100,000). The average annual dengue incidence rate was 172 (SD =93) per 100,000 population. The total number of dengue cases was 146,180 cases, and the total number of fatalities was 91. Figure 1b shows the box plot of the dengue incidence rate in Bangkok. The box encompasses 50% of the distribution, the line within the box is the median value, borderlines are the first, and the third quartile and small cycles are outliers. January, September, October, November, and December have outliers as illustrated in the figure.

### Decomposition

**Fig. 2:**
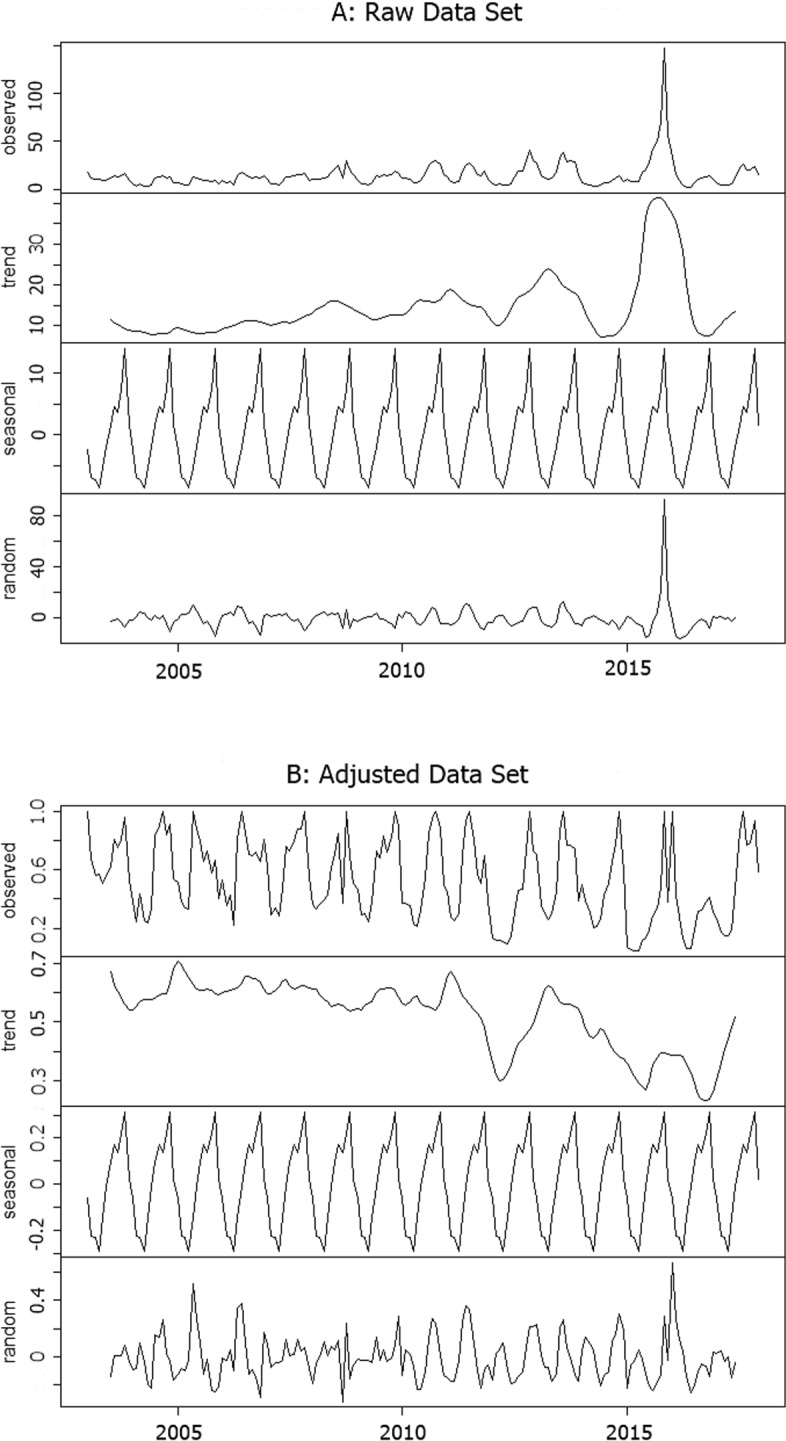
The decomposition plot of the time-series dengue case in Bangkok from 2003 to September 2017. **a**) The decomposition plot of raw data set; **b**) The decomposition plot of adjusted data set; The other layers show the decomposed components, representing the seasonal, trend, and random component, respectively

We created the adjusted data from the raw data by using Eq. (2). Figure 2 shows STL plot of two data sets, raw and adjusted data. Figure 2a shows SLT of the raw data set. There is a prominent high peak in the figure because the massive outbreak of dengue occurred in 2015. The trend and random component also clearly display this peak. Figure 2b displays the STL of the adjusted data. The maximum value of the dengue incidence rate was one. Therefore, extremely high peak or outlier incidence was limited to one. The configuration of the components of both data set is different, as can be seen in the figure.

**Fig. 3:**
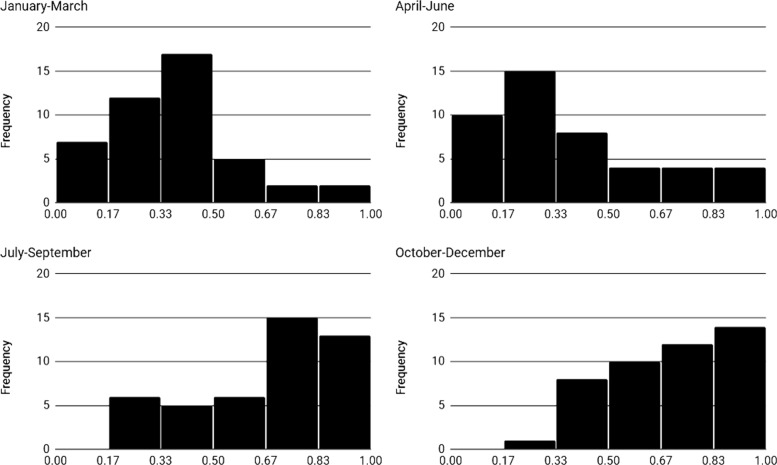
Histogram of adjusted dengue incidence data, $Y^{*}_{t}$ in Bangkok from 2003 to 2017, classified by quarterly

In this study, we evaluated the component that was the most critical component factor for the dengue-endemic in Bangkok. The ratio (*r*) values between the variance of each component and the variance of the data were calculated. For the raw data set, the ratio is 0.208, 0.281, and 0.443 for seasonal, trend, and random components, respectively. For the adjusted data set, the ratio is 0.455, 0.167, and 0.361 for seasonal, trend, and random components, respectively. Adjusted dengue incidence data in the first and second quarters of the year are right-skewed distribution, as illustrated in Fig. 3. The left-skewed distribution is in the third and fourth quarters of the year.

### Multivariate poisson regression model (MPR)

**Fig. 4:**
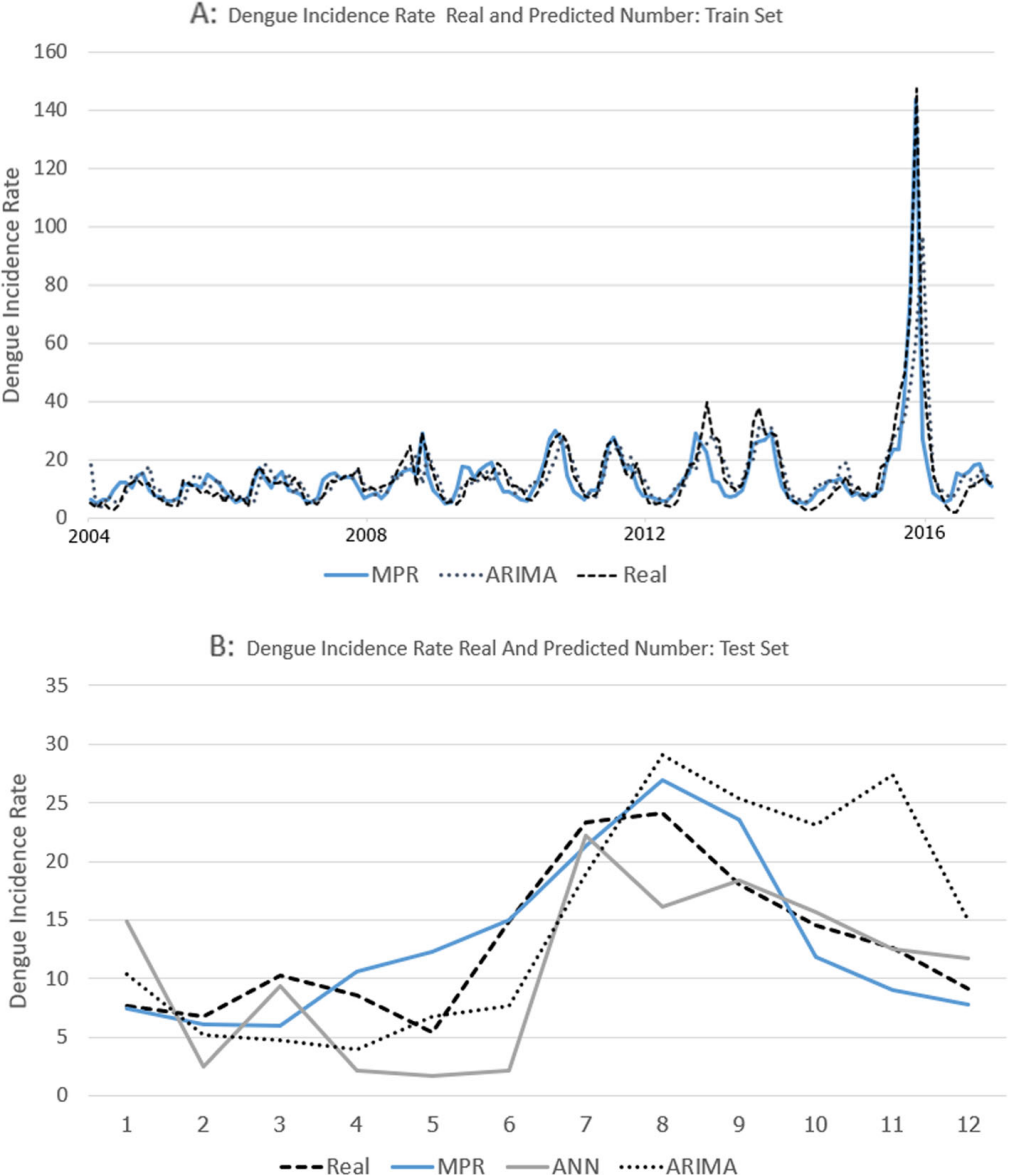
**a**: The dengue incidence rate per 100,000 population between real number in 2004-2016, the train set. **b**: The actual number in 2017 and the predicted number (MDR, ANN, and ARIMA), the test set

Table 1 shows the Spearman correlation analysis of the relationship between dengue cases (2003-2017) and climate variables with a time-lag of zero to three months. The positive regression was observed in humidity and precipitation, while the mean temperature was negative regression during the study period. The MPR model (lag 1-3 months) with the autoregressive term was established in this study by using the monthly climate data of Bangkok. The time lag 0 was excluded because the objective of the model aimed to predict the future number of dengue cases. The period 2003-2016 was the training set (168 samples), and 2017 was the test set (12 samples). After fitting the models for the training set, we used the model to predict the monthly dengue cases and compared to the test set. Table 1 displays the coefficient of the parameters and Fig. 4 illustrates the predicted dengue incidence cases.The correlation graph can be found in the correlation sheet in Supplementary file.

**Table 1:** Results of Spearman’s coefficient of rank correlation for time-lag effects, coefficient value, importance of independent variables

Climate variable	Time-Lag	MPR		ANN	
		Correlation	Coefficient	Importance	Normalized
			(× 10^−3^)		Importance (%)
Relative Humidity (%)	0	0.224	NA	0.228	68.1
	1	0.380**	9.167	0.069	68.1
	2	0.388**	1.735	0.093	91.6
	3	0.268**	-1.168	0.056	54.8
Rainfall (mm)	0	0.125	NA	0.162	82.8
	1	0.373**	1.333	0.087	85.9
	2	0.396**	0.651	0.102	100
	3	0.245*	0.200	0.083	81.7
Temperature (C ^∘^)	0	-0.150	NA	-0.096	62.2
	1	-0.077	-71.801	0.069	68.3
	2	0.144	11.606	0.079	77.9
	3	0.271*	5.869	0.059	58.2

The correlation of variables with intercept 1.11 × 10^−3^ and coefficient for previous case is 0.443 × 10^−3^. Spearman rank correlation and Pearson correlation analyses were performed with temperature and rainfall respectively.

*: *p*-value < 0.05, **: *p*-value < 0.01

### Artificial neuron network (ANN)

The ANN with one hidden layer and nine neurons was employed in this study. The number of neurons provided the least errors in this data set. The predictor importance number indicates the relative importance of each predictor or variable in estimating the model. The importance of an independent variable is a measure of how much the network’s model-predicted value changes for different values of the independent variable. Normalized importance is simply the importance values divided by the largest importance values and expressed as percentages. The results show that the importance of independent variables of climate factors to dengue cases. The highest was rainfall with two-month lag time, followed by relative humidity with the same lag time as display in Table 1. The predicted number of test values, as shown in Fig. 4.

### ARIMA model

We used the natural logarithm of dengue incidence for 2003-2016 as a test set. The best fit model was SARIMA (1,0,2)(1,1,2)^12^. In this time series, there was a strong seasonal component (1,1,2) and with the seasonal component (1,0,2), considered a mixed model.

### Performance of models

Figure 4a displays the plot between actual and predicted the number of dengue cases from the methods in the train set. The number of dengue cases in 2017 was used as a reference to test the accuracy of the results. Figure 4b shows a comparison between real data and predicted value. To obtain the most accurate method, we used several ways to evaluate the results. The correlation coefficient, Mean Absolute Error (MAE), Root-Mean-Square Error (RMSE), and Mean Absolute Percentage Error (MAPE) were a measure of prediction accuracy of a forecasting method that employed in this study. The MPR model achieved 0.87, 2.69, 3.37, and 26.41 for the correlation coefficient, MAE, RMSE, and MAPE, respectively. The ANN obtained 0.69, 4.07, 5.53, and 39.12 for the correlation coefficient, MAE, RMSE, and MAPE, respectively. Finally, the ARIMA was 0.90, 3.83, 6.49, and 26.45 for the correlation coefficient, MAE, RMSE, and MAPE, respectively. The results have shown that the MPR model has lower errors in every measurement compare to the others. The summary of the model comparison displays in Table 2. The total number of dengue cases in 2017 in Bangkok was 8781. The MPR, ANN, and ARIMA predicted the numbers of dengue cases were 8929, 7317, and 10038, respectively.

**Table 2:** Model Comparison

Model	Correlation Coefficient	MAE	RMSE	MAPE
MPR	0.87	2.69	3.37	26.41
ANN	0.69	4.07	5.53	39.12
ARIMA	0.90	3.83	6.49	26.45

## Discussion

The objective of this study was to evaluate the pattern of dengue incidence and the association between the number of dengue cases and climate factors in Bangkok (2003-2017). The unusual dengue-endemic was November 2015, which was nearly ten times the average number of dengue incidence rates in the study period, which may cause a significant error for prediction. Several outliers appeared in a boxplot. The outlier value may alter the accuracy of the model [14]. The adjusted data set assists us in exploring the pattern of the peak of the dengue incidence by reducing the effects of outlier value.

In this study, none of the elements has a ratio of variance exceed 0.5. Therefore, none of the components controls the pattern of dengue incidence of Bangkok. The ratio value (*r*) has shown that the random component was the most important to the raw data set. This result explains the appearance of outlier dengue cases. The seasonal component was the most crucial component of the adjusted data set. The peak of dengue cases may occur at varying times each year. Although the histogram of adjusted dengue incidence rate (Fig. 3) indicates that the peak time of dengue incidence in Bangkok is likely to be in the last quarter of the year.

Climate variables affect the mosquito population dynamics and disease transmission ability. In this study, the MPR and ANN model suggested that relative humidity and rainfall contribute to the impact on dengue transmission in Bangkok. The highest correlation was relative humidity with a two-month time lag, and the highest ANN importance was rainfall with also two-month time lag. Similar results also found in previous studies [8, 16, 17]. There was a difference in the distribution of dengue fever within and between provinces in Thailand [18, 19]. Increasing temperature contribute to a minor negative association because the mean temperature in Bangkok was relatively constant throughout the year. The average temperature during the study period was 29.8 ^∘^C (SD =1.29), which was close to the optimal temperature for dengue transmission, 29.3 ^∘^C [7]. The high temperature may decrease vector populations in warmer regions that are currently close to the limit for the mosquito to survive [20].

Rainfall generally increases the breeding sites for mosquitoes; its impact on dengue transmissibility was moderate in this study. There are plenty of human-made water containers such as jars, drums, pools, discarded tires that are mostly independent of rainfall in Bangkok. They become breeding sites for mosquito in the urban area. Also, stagnant water and poor sanitary and hygiene practices may make suitable breeding sites for the mosquitoes. In contrast, heavy rainfall may wash away breeding sites, interrupt the development of mosquito eggs or larvae [21]. The seasonal pattern indicates that the peak of the dengue-endemic in Bangkok usually occurs in November during the study period, which is generally outside the rainy season.

Typically, humidity increases the survival rate of mosquito and daily biting rates [22]. However, humidity above 79% may reduce the population of mosquito due to complex interactions between climate factors [23]. In Bangkok, the average humidity was 72.9% (SD =5.5) during the study period. Therefore, the humidity level in Bangkok is still in the condition that increases dengue transmission ability.

The models may be employed to predict the effect of the climate factors on the number of dengue cases. We used the most accurate MPR model to inspect the impact of changing the variables on the prevalence number. The MPR model showed that 1% rise of rainfall corresponded to an increase of 3.3% in the monthly incidence rate of dengue while 1% rise of humidity increase of 0.7% of dengue case in the model. However, a 1% rise of temperature corresponded to a decrease of 1.6% of dengue case.

Besides climate, there are many factors to consider. Several studies suggested that climate variables may contribute a minor effect to dengue transmission [13, 24]. In Singapore, urbanization is one of the main reasons for rapid dengue growth in the past 40 years [25]. Lee et al. calculated that none of the climate variables was a significant factor in the dengue transmission model for Ho Chi Minh City [11]. Johansson et al. stated that climate data did slightly improve the accuracy of the seasonal autoregressive dengue models for Mexico [13]. Female mosquitoes and seasons strongly correlated with the number of dengue cases in some provinces in Thailand [26]. Bangkok is one of the densest cities in the world, and it is likely to affect the pattern of dengue fever. Li et al. [27] found that urbanization may increase the abundance and survival rate of mosquitoes, which can increase the transmission ability and the number of infections.

There were several limitations to this study. Firstly, the actual dengue infection could be underestimated because persons who diagnosed with only mild or asymptotic symptoms usually not seek medical care. The real number maybe 4-6 times of reported cases [28]. Secondly, Bangkok is the center of economy and education. Every day, millions of persons travel to Bangkok in the morning and leave in the evening or early night. Some of the patients may be infected in Bangkok but obtained medical care somewhere else, which is hard to identify the place of infection. Therefore, human movement, urbanization, and transportations are essential factors to determine the dynamics of dengue transmission [29]. The non-climate variables may be added to the models if the data is available in the future. Another significant limitation is the effect of mosquito control programs excludes from the models. This factor may provide information on the potential of campaigns on the mosquito population control and may be useful in future researches. Instead of monthly data, the weekly or biweekly data may provide more detail about the nonlinear association between climate factors and dengue cases. However, BoE stores only monthly data on its online database.

Three models in this study displayed a different level of accuracy when compared to the test set. ANN showed a poor performance in predicting dengue cases compare to MPR in this study. The error in ARIMA was slightly above MPR. The results indicate that a single model may insufficient to predict the number of dengue because there are several factors that direct and indirect effects the transmission ability. This study provided three different approaches to forecast the number of dengue cases in Bangkok.

## Conclusion

The results have shown that the pattern of dengue in Bangkok relies only partially on the seasonal component. Rainfall and humidity have an impact on dengue transmissibility in Bangkok with a positive association. It is strongly recommended to add more variables to increase accuracy. These findings may be useful for developing climate models for dengue outbreak early warning method for Bangkok and the rest of the country.

## Supplementary information

**Additional file 1** Data set sheet contains the climate data in Bangkok form 2003-2017. **Table S1** is mean temperature. **Table S2** is rainfall. **Table S3** is humidity. **Table S4** is Dengue incidence number in Bangkok from 2003 to 2017. Correlation sheet contains the correlation between dengue case number and climate data in Bangkok.

## Data Availability

The data that support the findings of this study are available from the Department of meteorology of Thailand [15] and, Bureau of Epidemiology of Thailand [5].

## Abbreviations

ANN: Artificial neural networks
ARIMA: Autoregressive integrated moving average
DF: Dengue fever
MPR: Multivariate poisson regression
STL: The seasonal-decomposition procedure based on loess
